# Supplementary material for: Are carbon emissions trading and green financial instruments synergistic? -Comprehensive quantitative research based on content analysis
Source: PLoS One. 2024 Mar 7;19(3):e0298601. doi: 10.1371/journal.pone.0298601 (PMC10919601; doi:10.1371/journal.pone.0298601)
Supplement: S1 Data — (ZIP) [file pone.0298601.s001.zip › Data/地级市Z_F工作报告.zip!/║■─╧/╓Ω╓▐╩╨/└┤╫╘╣1⁄2╓┌║┼í╛┬φ┐╦╩2╛▌═°í┐_2016.docx]

各位代表：

现在，我代表市人民政府向大会报告工作，请予审议，并请各位政协委员和其他列席人员提出意见。

一、2015年及“十二五”时期工作回顾

2015年，是“十二五”的收官之年。这一年，在省委、省政府和市委的坚强领导下，我们积极应对新常态，扎实做好稳增长、促改革、调结构、惠民生、防风险各项工作，全市经济平稳健康发展，社会大局和谐稳定。

（一）精准发力稳增长，经济发展稳中有进。面对新常态下持续加大的经济下行压力，面对老工业城市转型调整的阵痛，我们保持定力，精准发力，千方百计稳增长，制定实施“稳增长43条”，开展“千家企业走访帮扶”活动，科学调度经济运行，较好地实现了全年预期目标。2015年，预计全市地区生产总值增长9.5 %，粮食生产实现“十二连丰”，农业增加值增长3.9%，规模工业增加值增长8.2%，社会消费品零售总额增长12.1%，一般公共财政预算收入、地方财政收入分别增长10%、14.3%，城乡居民人均可支配收入预计分别增长10%和11%。GDP、投资、消费、规模工业增加值等指标增速高于全国、全省平均水平，多项指标在全省排位前移。

（二）坚定不移调结构，产业培育彰显成效。优势产业取得突破。轨道交通产业收入突破1000亿元、圆了株洲“千亿产业梦”，北汽一工厂50万辆整车成功下线，航空产业产值增长62%。积极对接“中国制造2025”，全省首批入选国家“智能制造”的4个项目全部落户株洲，全市10个项目进入首批“制造强省”笼子。新兴产业来势看好。电子信息、新能源、新材料、生物医药等战略性新兴产业成为经济发展重要增长点，中国电子可控计算机项目开工建设，麦格米特、炎帝生物等一批企业快速成长。“互联网+”催生新业态，阿里巴巴株洲产业带年销售收入突破20亿元，炎陵“互联网+黄桃”电商模式全省推广，我市成为全国首批信息消费示范城市。创新优势不断凸显。国家自主创新示范区建设全面启动，株洲·中国动力谷自主创新园一期建成开园，中科院智能视频识别技术等15个项目成功入园，引进6位院士在园区设立工作站。中车株洲所两项科技成果在2015年全国科技奖励大会上，分别荣获国家科技进步特等奖和国家技术发明二等奖。深入实施创新创业园区“146”工程，全年建成标准厂房264万平方米，为企业“拎包入驻”提供了便利条件。建成了一批众创空间，大众创业、万众创新蔚然成风。

（三）攻坚克难扩投资，项目建设加速推进。大力推进“422”工程，全市完成社会固定资产投资2181亿元，增长18.7%。围绕优势产业、政策机遇，策划并启动轨道交通城、新马汽车博览园、城铁株洲站城市综合体等百亿项目建设。积极参加“港洽周”活动，成功举办深圳（株洲）招商推介会，长城电脑、深圳瑞丰光电等一批项目落户株洲。不断创新融资方式，走出了债券、基金、中票等多元融资新路子。全年新增直接融资246亿元，争取国家各类资金102亿元。投资35亿元的轨道交通城路网、铁东路核心段两个PPP项目开工建设。投融资体制改革经验在全国、全省大会上推介。大力推进征拆征收工作，完成集体土地征拆4.28万亩，同比增长33%；完成国有土地房屋征收62.07万平方米，其中市本级完成50.83万平方米，同比增长两倍。大力推进拆迁货币化安置，有效刺激住房消费，全年完成商品房销售面积658.6万平方米，增长22.6%，增速排全省第一。

（四）蹄疾步稳抓改革，市场活力竞相迸发。大力开展“改革深化年”活动，9大类65项重点改革稳步推进。推行建设投资项目审批改革，项目审批时间由过去的200多个工作日缩短到70个工作日以内办结。深化财政预算体制改革，归集财力11.3亿元，设立产业专项发展基金和偿债基金，加强财政资金绩效评估，资金管理成效显著。加快市级政府投融资平台公司改革。建立“借得到、用得好、还得起”的地方政府性债务管理机制，置换政府性债务80.7亿元，有效防范了债务风险。市县区政府机构改革全面完成，乡镇区划调整基本完成，国有林场改革通过省级验收。农村“三块地”确权颁证、不动产登记制度、公共资源交易管理体制等改革有序推进。与此同时，不断扩大对外开放，申报B型保税物流中心取得重要进展，成功获批铁路综合口岸。株洲企业开拓海外市场又有新进展，时代电气收购英国SMD深海机器人项目，旗滨玻璃在马来西亚投资建厂。全年完成出口15.5亿美元，增长18.3%。

（五）真心实意办实事，民生保障不断加强。深入实施“民生100”工程，持续加大财政投入，民生支出占公共财政支出的72%，过百项民生实事全面完成。坚持以创业促就业，全市新增城镇就业6.21万人、农村劳动力转移就业1.69万人。全面实行机关事业单位新的养老保险制度，建立城乡居民大病医疗保障制度。全年新建公租房1万余套，完成棚户区改造近2万户，农村危房改造9300户，解决27.21万农村人口饮水安全问题，“两房两棚”、“两供两治”工作经验在全省推介。教育质量不断提高，全市所有县市区都通过教育部“义务教育基本均衡县”评估验收，职业教育规范管理经验全国推广，职教园建设加快推进。临床医学重点专科建设三年行动计划有序推进，中南大学湘雅医学院附属株洲医院正式挂牌运营。成功入选“第三批国家公共文化服务体系示范区”创建城市。提前五年停止征收全市路桥车辆通行费。大力推进扶贫攻坚，实施扶贫项目372个，减少贫困村31个，6.16万人实现脱贫，城市五区建档立卡贫困人口全面脱贫。安全生产、计划生育考核获评全省先进市州。

各位代表，过去的一年，困难比预想的多，结果比预想的好。全市目标任务的顺利完成，标志着“十二五”圆满收官。

这五年，是综合实力明显增强的五年。全市上下集中力量打好“四大攻坚战”，实现了经济总量、人均均量和运行质量“三量齐升”。经济实力迈上新台阶。全市经济总量突破2000亿大关，2015年预计达到2350亿元，五年增长84.3%；财政收入达到290.4亿元，五年增长1.2倍；固定资产投资五年累计完成7522亿元，是“十一五”的三倍；规模工业增加值迈上1000亿台阶，达到1087亿元，五年增长79.6%；五年存贷款余额分别达到2412亿元、1234亿元，分别增长1.1倍和1.2倍。人均GDP、人均财政收入、城乡居民人均收入、小康实现程度均居全省第二。经济结构持续优化。三次产业结构由“十一五”末的9.7：57.8：32.5预计调整为2015年的7.5：58：34.5。“五城四基地”全面推进，株洲·中国动力谷迅速崛起，轨道交通、汽车、航空产业增加值年均分别增长10.8%、11%、30%。高新技术产业增加值占规模工业增加值比重达48.4%。株洲高新区在全国高新区综合排名从第40名跃升至第28名。预计非公有制经济占GDP比重达57%。县域经济占GDP比重提高到52%。经济质量和效益稳步提升。全市城乡人均可支配收入年均分别增长11.9%和15.8%，均高于GDP增速。全市规模工业企业利税实现翻番。万元规模工业增加值能耗累计下降62.9%，大大超过“十二五”节能减排目标任务。

这五年，是城乡面貌变化最大的五年。大力推进以人为核心的新型城镇化，城乡一体化迈出新步伐，全市城镇化率由55.5%提高到62%。城镇发展空间不断拓展。市区建成区面积由“十一五”末的112平方公里扩大到138平方公里。全市城镇建成面积由“十一五”末的255平方公里扩大到337平方公里。城乡基础设施明显改善。“十二五”全市完成基础设施投资1770亿元，是“十一五”时期的3.8倍。沪昆高铁、衡茶吉铁路和平汝、垄茶等高速公路建成通车，形成了“四纵三横”交通新格局，实现了“县县通高速、县县通铁路”。累计完成农村公路建设2700公里，其中新建1345公里，全市农村公路总里程突破1万公里。长株潭一体化进程加快，城际铁路及5个站场建设加快推进，3条城际干线公路基本通车。神农城、河西湘江风光带、云龙大道、荷塘大道等一大批城市重点基础设施工程建成，拉开了城市框架，增强了城市功能，提高了城市品位。完成504个村的农网改造，全省首个垃圾焚烧发电厂投入使用，生活污水和生活垃圾处理率明显提高。除险加固病险水库485座，解决了101万农村人口安全饮水问题，建成高标准农田72.8万亩。城乡管理模式持续创新。探索形成“运营市场化、管理网格化、作业精细化、考核标准化”的城市管理模式，实现了由政府包揽向市场运作、突击式管理向常态化管理、城区为主向城乡同治转变。加快建设“智慧株洲”，全面建成治安防控、智能交通、数字城管、数字环保“四大平台”。成功创建全国文明城市，顺利通过国家卫生城市复审、全国交通管理模范城市复评，荣获“中国人居环境范例奖”。城乡环境大为改观。湘江保护与治理省政府“一号重点工程”有力推进，清水塘老工业基地搬迁改造取得重要进展，“三个基本行动”成效显著。全市关停企业473家，淘汰落后生产线195条，拆除烟囱452根，关停养殖企业529家，湘江株洲段水质保持国家Ⅲ类水质标准，市区空气质量优良天数逐年增加，2015年达到281天。攸县城乡环境同治经验在全国推广，农村生产生活环境极大改善。全面推行封山育林，成功创建国家森林城市，全市森林覆盖率提高到61.9%。

这五年，是发展动力加速转换的五年。坚持改革开放创新，新的发展动力正在加快孕育和形成。改革红利不断释放。在全省率先公布权力清单、责任清单、涉企收费清单和外商投资准入管理目录，市本级行政审批事项由302项减至122项，向园区下放权力62项，全面取消非行政许可审批事项；着力改革商事制度，实行注册资本认缴制、“三证合一、一照一码”，市场主体成倍增长。推行“两集中、两到位”，优化审批流程，缩短审批时限，实行绿色通道、容缺受理、全程代办等特色服务，获得李克强总理肯定。全面完成两型社会试验区第二阶段改革建设任务，两型社会建设走在全省、全国前列。成立株洲市农村集体土地流转市场，推进城乡建设用地增减挂钩。市属国有工业企业改革基本完成，央企“三供一业”改革经验全省推广。全口径政府预算体系基本建立，严格执行国家结构性减税政策，稳步推进“营改增”。农村信用社等金融领域改革取得明显成效。对外开放成效显著。加大“引进来”力度，累计引进外资35.3亿美元，引进内资1150.9亿元。加快“走出去”步伐，以神农文化为主题，不断加强对台交流。中车株机、中车株所等一批企业走出国门实行海外并购、建立多个海外基地。累计境外投资4.7亿美元、进出口总额118.9亿美元。出口从单一输出“产品”向输出“产品+服务+投资+技术”转变，逐步形成了多点支撑、多方带动的开放格局。

这五年，是群众得实惠最多的五年。坚持以“十大民生实事”、“民生100”工程为抓手，集中力量办成了一批实事好事，民生民利持续改善。全力织牢基本民生“保障网”。建成湖南（株洲）创业就业指导中心，成功获评“全国创业先进城市”。大力推进保障性住房建设，城镇保障性住房覆盖率从“十一五”末的5.6%提高到21.9%，处于全省领先水平。实行城乡居民养老保险制度并轨，推进城乡医保、低保扩面提标，覆盖城乡的社会保障体系基本建成。努力扩大社会事业“普惠面”。在全省率先实现每个乡镇（街道）建成一所公办幼儿园、义务教育学校全部建成合格学校，市二中搬进新校址办学；职教园签约入园职业院校10所，其中5所建成开学，教育强市建设迈出可喜步伐。公立医院改革纵深推进，全面实行基本药物制度和药品零差额销售，在全省率先破除“以药补医”，新农合参保率稳定在97%以上，“看病难”、“看病贵”的问题得到有效缓解。“三馆三中心”加快建设，社区文化活动中心、乡镇综合文化站、农家书屋建设全面完成。合力构建和谐稳定“大格局”。深入推进法治株洲、平安株洲建设，扎实开展“三调联动化矛盾、息诉息访促平安”专项调解活动和化解信访积案专项行动，获得全国综治工作优秀地市“五连冠”、民族团结进步“六连冠”，三次捧得“长安杯”。市县乡村四级安全生产监管体系全面建立，安全生产形势平稳可控。食品药品安全监管城乡一体化建设扎实推进。深入开展军民共建，实现“全国双拥模范城市”五连冠。不断加强国防动员工作，驻株解放军、武警部队和民兵预备役，为全市经济社会发展作出了积极贡献。同时，计生、统计、人防、移民、国安、质检、供销、气象、广电、新闻出版、体育、档案、地方志、对口支援、妇女儿童、残联、贸促等工作都有新进步。

这五年，是政府自身建设切实加强的五年。坚持转职能、改作风、提效能，打造法治型、服务型、效能型、廉洁型政府，政府治理能力不断提升。严格落实公众参与、专家论证、风险评估、合法性审查、集体讨论决定等重大决策程序，有力推进民主科学决策。制定出台政府工作规则、政府投资项目管理等制度性文件，政府权力配置和运行边界进一步规范。市长热线已经成为政府与广大市民的“连心线”，政务公开工作全面加强。依法治市工作取得明显成效。认真执行人大及其常委会决议、决定，自觉接受人大、政协和社会各界的监督，五年共办结人大代表建议1461件，政协委员提案1979件，问题解决率不断提高。深入开展群众路线教育实践活动和“三严三实”专题教育，严格执行作风建设有关规定，廉政建设和反腐败斗争取得积极成效，行政效能和服务水平明显提升。

各位代表，五年的成就令人振奋，积累的经验弥足珍贵。我们深切地体会到：做好政府工作，必须牢牢把握战略机遇这个发展高点，在全球、全国视野中谋划发展，积极对接国家战略，抢占发展先机；必须牢牢把握加快发展这个第一要务，以问题为导向，保持定力，精准发力，以“钉钉子”精神一抓到底；必须牢牢把握改革创新这个核心动力，破除体制机制障碍，破除思维定势、工作惯性和路径依赖，持续为经济社会发展注入新的活力；必须牢牢把握转型升级这个主攻方向，推动经济发展由依靠要素驱动向创新驱动转变，由注重量的增长向注重质的提升转变；必须牢牢把握民生改善这个根本目的，让改革发展成果更多更公平地惠及全市人民；必须牢牢把握改进作风这个重要路径，转作风、促政风、带民风，提升政府工作的执行力和公信力。

各位代表，历尽天华成此景，人间万事出艰辛。五年来，发展的成绩来之不易，这是市委正确领导的结果，是市人大、市政协监督与支持的结果，饱含了各位代表、各位委员的智慧和力量，凝聚了全市人民的心血和汗水。在此，我代表市人民政府，向全市广大干部群众，向人大代表、政协委员，向各民主党派、工商联、无党派人士和人民团体，向驻株部队官兵以及中央、省驻株单位，向离退休老同志，向所有关心支持株洲发展的各界朋友，表示崇高的敬意和衷心的感谢!

在总结成绩的同时，我们也清醒地看到，我市经济社会发展中还存在不少困难和问题：一是稳增长调结构压力仍然较大。我们既要面对新常态下经济下行持续增大的压力，又要面对湘江保护治理、推进清水塘老工业基地搬迁改造带来的阵痛；既要稳增长，又要调结构；既要去产能，又要惠民生；既要推改革，又要防风险；任务很重，压力很大。二是“四块短板”依然突出。县域经济实力不强，非公经济比重不大，现代服务业水平不高，开放型经济发展不快，成为全面建成小康社会不容回避的问题。三是影响社会和谐稳定的矛盾和问题仍然较多。财政收支矛盾、金融债务风险、安全生产、信访维稳等方面的问题不容忽视。四是脱贫攻坚难度大、时间紧，群众就业、就学、出行和住房等方面的难题仍然较多。五是发展环境有待进一步优化。适应新常态的体制机制尚未建立，“为官不为”和乱作为的现象还在一定程度上存在。对此，我们将高度重视，认真研究，采取有力措施，切实加以解决。

二、“十三五”时期的发展目标和任务

各位代表，“十三五”是株洲转型升级的关键期、新型城镇化的加速期、深化改革的攻坚期、全面建成小康社会的决胜期。

察势者智，驭势者赢。唯有把握大趋势、大逻辑，才能抢抓大机遇、赢得大发展。当前，全球经济在深度调整中曲折复苏，新一轮科技革命和产业变革蓄势待发；我国经济进入新常态，但经济发展长期向好的基本面没有变。我市既处于可以大有作为的重要战略机遇期，又处于爬坡过坎的关键期，发展方式正在加快转变，新的发展动能正在加速孕育。我们要始终保持开拓进取的锐气和昂扬向上的士气，努力在新常态中抢抓新机遇，实现新发展。

“十三五”期间政府工作指导思想是：高举中国特色社会主义伟大旗帜，全面贯彻党的十八大和十八届三中、四中、五中全会精神，以马克思列宁主义、毛泽东思想、邓小平理论、“三个代表”重要思想、科学发展观为指导，深入贯彻习近平总书记系列重要讲话精神，坚持“四个全面”战略布局，贯彻“五大发展”新理念，全面落实省委“三量齐升、五化同步”新要求，坚持发展第一要务，坚持以提高发展质量和效益为中心，坚持创新改革开放投资“四轮驱动”，加快形成适应经济发展新常态的体制机制和发展方式，以转型升级为总战略，以“三个率先”为总目标，实施“七大工程”，建设“四区一带”，统筹推进经济建设、政治建设、文化建设、社会建设、生态文明建设和党的建设，建设富强美丽幸福新株洲。

为落实这个要求，结合株洲实际，我们编制了我市“十三五”规划纲要，提请大会审查。“十三五”全市经济社会发展的主要目标是：GDP年均增长9%左右；一般公共财政预算收入年均增长9%以上；全社会固定资产投资年均增长16%；社会消费品零售总额年均增长12%；城乡居民收入分别年均增长9%、10%。各项事业都有新的发展。

实现上述目标，必须突出抓好以下五个重点：

（一）坚持创新发展，培育经济发展新动能。全面深化改革，在供给侧和需求侧两端同时发力，扩大有效供给，创造新的需求，激发新的动能。以科技创新为引领，大力实施创新创业工程，建设国家自主创新示范区，促进大众创业、万众创新。积极对接“中国制造2025”、“互联网+”行动，大力实施产业振兴工程，推动产业向中高端迈进，建设先进制造业集聚区。全力打造株洲·中国动力谷，将株洲建设成为辐射全球、国内一流的动力之都。大力发展“四新”经济，重点开发3D打印、物联网等新技术，发展智能工业机器人、大数据应用等新产业，培育科技金融、文化创意等新业态，构建制造业与服务业融合发展新模式，走出一条创新转型发展的新路子。

（二）坚持协调发展，拓展城乡发展新空间。以国家新型城镇化综合试点市为抓手，大力实施城镇扩容提质工程，积极推进智慧城市、海绵城市建设，建成现代化国际化生态宜居城市。构建“一核一圈一廊”空间布局，做强中心城区核心增长极，打造株醴都市圈，建设醴炎县域经济走廊，加快形成中心城市、次中心城市、县城、中心镇协调发展、互促共进的现代城镇体系。以户籍制度改革等五大试点任务为重点，加快推进以人为核心的新型城镇化，促进城乡协调发展。坚持“五位一体”、“五化同步”，推进县域崛起工程，打造平汝高速沿线新的经济增长带。深化文明创建，促进物质文明与精神文明协调发展。积极支持国防建设，促进军民融合深度发展。

（三）坚持绿色发展，建设两型生态新家园。实施两型示范工程，率先在全国建成两型社会示范区。实行最严格的环境保护制度，严守环境质量底线、生态保护红线、资源消耗上限，合理布局生产、生活、生态空间，形成人与自然和谐的绿色发展新格局。用绿色倒逼产业升级，培育绿色产业，发展绿色经济。大力开展大气、水、土壤污染防治行动。加快推进清水塘老工业基地搬迁改造，深入推进湘江保护与治理省政府“一号重点工程”，抓好“一江两水四港”综合治理，推进山水林田湖生态保护和修复工程，筑牢绿色生态屏障。倡导绿色低碳、文明健康的生活方式和消费模式。深化生态文明体制改革，构建产权清晰、多元参与、系统完整的生态文明制度。

（四）坚持开放发展，打造合作共赢新优势。放眼全球，全域开放，全面对接融入国家“一带一路”、长江经济带战略，在湖南“一带一部”区域战略新定位中，立足株洲位于京广、沪昆两大经济带交汇的区位优势，打造湘东开放型经济带、湘赣开放合作带和国家产业转移重要承接带，成为中部崛起的战略支点。深入实施“引进来”、“走出去”战略，开展引资引技引智升级行动和对外贸易提升行动，营造市场化、法制化、国际化的营商环境，提升对外开放能力和水平。

（五）坚持共享发展，满足群众生活新期待。实施民生共享工程，创新提供方式，增加公共服务，保障基本民生，率先在全省全面建成小康社会。把脱贫攻坚作为第一民生工程来抓，实施精准扶贫工程，坚决打赢脱贫攻坚战，确保2017年现行标准下农村贫困人口全部脱贫，贫困县全部“摘帽”。坚持“普惠性、保基本、均等化、可持续”方向，加强义务教育、就业服务、基本医疗、社会保障、公共文化、环境保护等基本公共服务，让广大人民群众在共建共享中有更多的获得感。大力推进健康株洲建设，提高人民健康水平。健全社会治安防控体系，维护和谐稳定，建设平安株洲。

三、2016年总体要求和工作部署

千里之行，始于足下。实现“十三五”各项发展目标和任务，首先要做好2016年的工作。我们必须贯彻中央、省委和市委经济工作会议精神，坚持稳中求进、稳中有为的工作总基调，突出供给侧结构性改革，突出创新改革开放投资“四轮驱动”，突出统筹城乡协调发展，突出保障改善民生，深入开展“项目攻坚年”和“企业帮扶年”活动，促进经济持续健康较快发展和社会和谐稳定，实现“十三五”经济社会发展的良好开局。

今年全市经济社会发展的主要预期目标是：GDP增长9%以上；规模工业增加值增长8.5%以上；一般公共财政预算收入增长9%以上；全社会固定资产投资增长18%；社会消费品零售总额增长12%；城乡居民收入分别增长9%、10%；万元GDP能耗下降3.5 %以上。

围绕今年的目标任务，我们将重点抓好以下八个方面的工作：

（一）以创新驱动为引擎，大力推进产业振兴

加快建设制造强市。紧紧抓住“中国制造2025”行动的重大机遇，全力打造株洲·中国动力谷，加快建设“三基地一中心”。着力推进轨道交通城建设，加快推进中车大道等轨道交通城路网建设，加快中车在株三大研究院的建设，全力推进IGBT器件产业化和国内城际动车组产业化，向轨道交通产业2000亿元台阶迈进。着力推进通用航空城建设，加快通用机场大道、千亿大道等路网和中航工业南方公司新园区建设，加快小飞机整机及航空零部件制造项目建设。着力推进新能源汽车城建设，加快北汽二工厂、海纳川汽车零配件产业园等项目建设。培育壮大电子信息、新材料、新能源、健康食品与生物医药、节能环保等五大战略性新兴产业。做大做强服饰、陶瓷等传统产业。全面实施质量强市战略。大力推动智能制造示范。大力推进创新创业园区“146” 工程，加快标准厂房建设和基础设施配套，加大项目招商落地力度，不断提升园区实力。

精心打造创新之都。科技引领未来，创新成就梦想。全力推进国家自主创新示范区建设。加快落实已经出台的“6+4”等有关政策，进一步构建人才引进激励、科技成果转化、资源开放共享、军民协同创新、科技金融结合等政策体系，充分调动包括科技人才、企业家在内的各类人员创新的积极性。强化创新平台建设，打造产学研多方协同建设的重点实验室、工程技术中心、企业技术中心等一批研发平台，加快建设大功率交流传动系统集成、新型功率半导体器件等国家重点实验室。推动研发平台、检测平台、信息平台等各类创新平台对外开放、资源共享。聚焦三大动力产业、五大战略性新兴产业和传统优势产业，前瞻部署和实施一批重大科技攻关项目，实行企业技术需求和科技成果转化清单制度。支持企业技术改造和设备更新。加强知识产权保护。坚持军民融合发展，充分发挥608所、中航工业南方公司等株洲军工企业作用，建设军民融合产业基地，实现军民融合发展新突破。大力推进大众创业、万众创新。推行众创、众包、众扶、众筹新模式，加快建设一批众创空间，做大做强创客经济，让创新创业的涓涓细流汇聚成推动株洲发展的强大洪流。

加快推进现代服务业发展。大力发展金融保险、现代物流、职业教育、研发设计、信息服务、商务咨询等生产性服务业，加快发展商贸流通、文化旅游等生活性服务业，大力推进中车物流园、铁路物流园、株百天元城、市农产品交易中心、方特二期、茶祖园等项目建设。围绕株洲独特的炎帝文化、红色文化、工业文明和生态资源，建设一批精品景点景区，完善旅游服务体系，加强湘赣旅游合作，打造大湘东旅游带。搭建资源集中、发展集约的发展平台，加快中心商务区等九个服务业集聚区建设。加快打造一批信息服务平台、电子商务平台和物流配送平台，为服务业延伸发展提供有力支撑。深化与国内知名电商的战略合作，推动株洲产品“网上行”，加快“智慧株洲”建设，利用云计算、大数据平台，提升医疗、教育、交通、旅游、金融、物流、快递、零售等领域的在线化、智能化水平。

（二）以供给侧改革为核心，培育壮大市场主体

大力推进简政放权。继续取消和下放审批权限，深化建设投资项目审批改革，推进工商注册登记全程便利化，最大限度减少政府对微观经济的干预。注重放管结合、优化服务，加强事中事后监管服务，采取随机抽查、突击检查等方式，建立第三方评估监管监测机制，提高监管效率和质量，真正做到政府放权、市场发力。放宽社会资本准入领域，降低创新创业门槛，积极引入社会资本参与公共领域项目建设，以更大的力度推广PPP模式，支持和引导非公经济加快发展。

深入开展“企业帮扶年”活动。着力去产能、去库存、降成本、优环境。一是去产能。综合运用市场、行政、经济和法制手段化解产能过剩，健全市场化退出机制，对不符合环保、质量、安全标准且长期亏损的“僵尸企业”，采取兼并重组、破产清算等方式，分类有序予以“出清”。二是去库存。采取政府采购、支持企业拓展市场等措施，帮助企业减小库存压力。重点加快农业人口市民化，推进以购租并举为主要方向的住房制度改革，加大棚改货币化安置力度，化解房地产库存。三是降成本。清理规范中介服务，实行收费减免和税收优惠，降低企业用工、物流、财务和电力成本，将企业减负落到实处。四是优环境。深入实施工业企业“十百千”和商贸企业“1511”工程，精心打造“市长热线—企业服务专线”服务平台，创新服务方式，落实惠企政策。多措并举培育企业主体，通过增强现有企业竞争力改造提升一批，发挥龙头企业引领作用带动一批， 加大“个改企”力度升级一批，加快对外招商引进一批，推进大众创业、万众创新催生一批，形成市场主体如雨后春笋般不断涌现、发展活力竞相迸发的生动局面。

（三）以扩大需求为抓手，全力推动经济增长

大力开展“项目攻坚年”活动。把项目建设作为稳增长、调结构、惠民生的第一抓手。全年实施重点项目200个以上，带动全社会投资2500亿元以上。围绕项目来源抓前期。紧盯国家和省里“十三五”发展重大战略、重大工程、重大行动、重大资金、重大试点，结合我市的重大优势、重大潜力、重大短板，精心策划开发一批重点项目，加快推进前期工作，全力向上争取一批项目。要推广以商招商、以情招商、以链招商、以订单招商、以服务招商、以联盟招商等新模式，要特别重视异地商会的作用，以“不在招商现场就在招商路上”的作风，积极向外引进一批项目，确保招商引资有大的突破。围绕要素瓶颈抓突破。继续保持征地拆迁的工作力度，依法征拆、阳光征拆，确保项目用地。抓住当前融资环境相对宽松的机遇，千方百计筹集建设资金，确保资金供给。围绕建设进度抓协调。进一步创新项目工作推进机制，以“三严三实”的作风推进项目建设，强化责任分工、强化督查考核、强化责任追究，全面提高项目的开工率和竣工率。

释放消费需求。培育消费新热点。完善供应体系，激活房地产、汽车等改善性需求；加快国家信息消费示范城市建设，扩大信息消费；大力推广应用新能源汽车、绿色家电、绿色建筑等节能低碳绿色产品，倡导绿色消费；大力发展健康养老、家政服务、休闲旅游、终身教育、文化体育等新兴消费。营造消费新环境。加强市场监管，严厉打击损害消费者权益行为，从源头、渠道和终端上确保消费者放心消费，努力打造株洲消费品牌，让本地消费留下来，把外地消费引进来。

挖掘出口潜力。充分利用国家汇率改革和“一带一路”倡议机遇，创新出口方式，推进区域通关一体化，巩固轨道交通装备、硬质合金、陶瓷、烟花等传统特色产品出口，培育服饰、航空、汽车等外贸新增长点，力争全年实现出口增长10%以上。

（四）以改革开放为动力，不断激发发展活力

深化重点领域改革。深化行政管理体制改革，有序推进事业单位分类改革，实现政事分开、管办分离。加快产权制度改革，推进农村集体土地承包经营权、宅基地、集体经营性建设用地确权颁证，实施不动产统一登记制度，实现市县两级统一登记发证。加快公共资源交易体制改革，将涉及公共领域的资源交易项目统一纳入市公共资源中心交易，构建公正公平公开的公共资源交易体系。深化国资国企改革，完成政府投融资公司新一轮改革，加快处理上一轮国有企业改革遗留问题。深化财税体制改革，出台配套文件，完善全口径预算分配、财政运行综合绩效考评、债务预算管理和风险防控机制，编制政府投资项目债务预算规划，做好政府存量债务置换工作，建立跨年度预算平衡机制，理顺市区两级财政体制。深化金融体制改革，推进金融产品和服务创新，完成城区、茶陵县、株洲县农商行组建挂牌，支持攸县、炎陵县设立村镇银行，推动小额贷款公司和融资性担保机构健康发展，稳步发展产业发展基金等新兴金融业态，切实防范金融风险。开展农村“三权”抵质押贷款试点，着力解决“三农”融资难、融资贵问题。统筹推进其他领域改革。

打造对外开放新平台。加快铁路口岸和保税物流中心（B型）建设，争取湘欧快线延伸至株洲，积极申报株洲国家级经开区，建设铁路、公路、水运、保税物流、贸易通关于一体的综合性双向开放平台。加快实施“走出去”战略，推动跨境电子商务发展，扩大电子商务在外贸中的应用。推进国际产能和装备制造合作，引导企业“抱团出海”、“借船出海”，在互动合作中提升株洲制造业的实力和水平。

（五）以国家综合试点为契机，加速推进新型城镇化

推进“多规融合”，优化城镇空间布局。按照“一核一圈一廊”空间结构，加快完善或制定相关规划，推进城市总体规划、土地利用规划和经济社会发展规划相衔接，切实抓好炎陵县等地“三规合一”试点。做强中心城区核心增长极，以“北联、东拓、南进”为导向，加快建设云龙新城、东部新城、南部新区，构建“一体三极”城市空间形态。加快武广高铁新城、枫溪新城等一批城市综合体开发。打造株醴都市圈，加快莲易高速、东城大道建设，加快株洲县撤县设区和株醴融城步伐，建设株醴都市区创新发展试验区。建设醴炎县域经济走廊，以岳汝高速、106国道为纽带，做强南部三县，支持攸县撤县建市，加快建设市域副中心城市，加快茶陵、炎陵城镇化步伐。抓好攸县、白关镇、白兔潭镇等国家、省级城镇化试点工作，以及三门镇等16个特色镇试点工作。

织好“五张网”，完善基础设施体系。交通网，开工建设株洲火车站改造等一批项目，加快建设湘江大道、铁东路等一批项目，建成长株潭城际铁路及配套工程、湘江六桥、七桥等一批项目。实施交通堵点畅通工程三年行动，加快“公交都市”创建步伐，加强农村道路提质改造，积极争取渝长厦高铁采用“长沙—湘潭—株洲”通道，加快有轨电车前期工作，构建便捷的内外交通网络。水利网，开展全国水生态文明城市试点、全国河湖管护体制机制试点，推进一批重点水利工程建设，加快饮用水第二水源前期工作，解决农村16.6万人饮水安全问题，加快城镇防洪减灾能力建设。能源网，攸县煤电一体化项目投产发电，加强电力、燃气等基础设施配套，加快“气化株洲”建设，推进管道燃气向攸县、茶陵和炎陵延伸。信息网，推进“智慧株洲”建设，充分挖掘政府大数据的价值，打造全域化、多功能的信息应用平台，加快建设一批信息化应用工程。城镇配套网，加快推进市第四水厂提质改造和枫溪污水处理厂建设等一批“两供两治”工程，加强公交站场、地下综合管廊等地上地下硬件设施建设。实施城市绿荫三年行动计划，让市民开窗见绿、出门见景。

落实“四化”要求，提升城镇管理水平。贯彻中央城市工作和城镇化工作会议精神，推进城镇管理法治化、市场化、信息化、标准化。积极推进城市管理与行政执法体制改革，创新和加强城市综合执法。完善城市管理市场运营模式，逐步将灯饰管护、渣土清运等领域向市场放开，提升市场化运作水平。加强城市管理数字化平台建设和功能整合，建设综合性城市管理数据库，提升城市管理信息化水平。加快户籍制度改革，建立城乡统一的户口登记制度，提高户籍人口城镇化率。探索建立农业转移人口市民化成本分担机制，加大就学、养老、就医等基本公共服务投入力度，让市民化的人口享受更多的核心公共服务。提高市民素质，巩固文明城市成果，展示文明城市风采。

（六）以融合发展为路径，着力促进县域崛起

加快农业现代化进程。推进农业发展方式转变，积极走一、二、三产业融合发展的路子，让农业强起来、农村美起来、农民富起来。坚守耕地红线，加强高标准农田建设，稳定和提高粮食产能。引入产业链、价值链等现代产业组织方式，大力发展规模农业、科技农业、品牌农业、安全农业，构建现代农业产业体系。鼓励推动“依法自愿有偿”土地流转，发展适度规模经营。以“十百千”工程为抓手，加强职业培训，继续实施“万名职业农民培训三年计划”，加大政策惠农、金融扶农、科技兴农、“互联网+”强农的力度，扶持壮大家庭农场、专业大户、农民专业合作社、农业产业化龙头企业等农业经营主体，推动粮油、养殖、竹木、饲料、农产品加工等五个百亿产业加快发展。加强农业面源污染治理，深入实施耕地重金属污染防治，强化农产品质量安全监管，增强农业可持续发展能力。加大涉农资金的投入和整合力度，发挥财政投入的引导作用，撬动更多社会资金投入农业农村。

加快县域产城融合进程。实施产业强县，提效农业，提速工业，提升第三产业。推进特色发展，支持县市立足各自优势，发展陶瓷、烟花、能源、农产品加工等传统产业，以及文化旅游、健康养老等新兴产业。推进一体发展，围绕轨道交通等重点产业，加快产业链向县域延伸，推进清水塘老工业基地中符合环保要求的企业向县域转移。推进承接发展，大力推进园区建设，主动承接发达地区产业转移，积极引老乡、回故乡、建家乡。围绕产业发展，打造一批工业重镇、文化古镇、旅游名镇、边界要镇。加快新农村建设进程，开展农村人居环境“千村整治”行动，大力实施美丽乡村“一廊十片百村”示范工程。

加快脱贫攻坚进程。着力解决“扶持谁、谁来扶、怎么扶、如何退”的问题。按照“四个切实”、“五个一批”、“六个精准”的要求，深入实施产业扶贫、基础设施、素质提升、群众增收、易地搬迁、兜底保障等六大工程，切实抓好农村危旧土坯房改造、纯农户大学生就业清零、关爱农村留守家庭三件实事。积极推进产业扶贫，立足特色资源禀赋，宜农则农、宜工则工、宜游则游，因村因户，分类指导，以产业带就业，以就业促脱贫。积极推进基础设施扶贫，加快水、电、路、气、房、环境治理“六到农家”。积极推进素质提升扶贫，充分利用株洲职业教育资源丰富的优势，全面完成6300名贫困对象的劳动技能培训。积极推进易地搬迁扶贫，对“一方山水养不起一方人”的贫困户，利用难得的政策机遇，加快推进易地搬迁脱贫。积极推进兜底保障扶贫，扎实抓好就医、就学、养老、低保、五保“五个保障”，实施“雨露计划”，落实贫困地区“两免一补”、学生“营养餐”计划、专项招生计划、助学专项补助基金等政策。加大扶贫资金投入，加强资源资金整合和监管，做大金融扶贫平台，提高扶贫资金实效。大力推进电商扶贫。抓好驻村帮扶，引导各类企业、社会组织、个人参与脱贫攻坚，构建专业扶贫、行业扶贫、社会扶贫“三位一体”的工作格局。确保全年减贫4.98万人，实现60个贫困村摘帽。

（七）以绿色发展为引领，加快两型社会建设

加大湘江流域保护和治理力度。按照“治调并重，建管结合”的要求，全面启动湘江保护与治理第二个“三年行动计划”。加快湘江河东段综合治理工程建设，深入推进“一江两水四港”环境综合治理，全面完成市区37家洗水企业整体搬迁入园。全面推进城乡环境同治，加快城镇生活污染处理，不断削减城镇生活污水和垃圾排放量，稳步提高湘江流域株洲段水质，努力朝着“江水清、两岸绿、城乡美”的目标迈进。

加大清水塘老工业基地搬迁改造力度。按照“一年初见成效、三年大见成效、五年完成综合治理、十年建成新城”的总体目标，完成污染治理规划和生态新城规划编制。采取“引促结合”、“分类支持”的措施，关停一批重点污染企业，力争完成一批重点企业的新址选址开工，部分企业进入新址标准厂房生产经营。开展环境污染第三方治理，启动搬迁企业污染场地治理，确保清水塘老工业区搬迁改造初见成效。

加大生态文明制度建设力度。全力推进两型社会试验区第三阶段改革建设工作，全面实施生态文明体制改革“1+6”方案，严格落实环境保护责任，构建源头预防、过程控制、责任追究的生态文明制度体系，推进废水、废渣、废气和重金属土壤整治项目，用制度保护环境、节约资源。推广应用两型技术及产品，充分发挥政府采购作用，引导培育节能环保产业，促进形成绿色生产方式和消费方式。建立工业用地节约集约利用机制，开展园区土地评价工作，提高工业用地产出效益。建立生态文明联动执法机制，加强生态文明建设考核，健全大环保格局。开展两型标准化建设，积极推进示范创建，总结推广一批两型示范模式。

（八）以民生“100”工程为载体，切实增强群众获得感

不断完善社会保障体系。突出抓好高校毕业生、就业困难人员等重点群体就业，确保全年新增城镇就业5万人，农村劳动力转移就业1.5万人。全面实施机关事业单位养老保险制度改革，按政策提高企业和机关事业单位退休人员养老金。深入推进全民参保登记计划，将更多的人纳入社会保障。健全低保标准增长机制，逐步实现农村低保和贫困标准融合。加强住房保障，改造各类棚户区1.97万套。

加快建设“健康株洲”。深化医疗卫生体制改革，实施分级诊疗制度，提升乡镇中心卫生院和村（社区）卫生机构的服务能力。加快公立医院改革，实施临床医学重点专科建设三年行动计划。全面落实两孩政策，改革和完善计划生育服务管理，支持开展出生缺陷干预工作，提高计生特殊家庭补助标准。实施食品安全战略，建立食品安全追溯系统，形成覆盖生产、流通、消费全过程的监管机制，启动食品安全城市创建。大力推动各类体育设施对社会开放，深入开展全民健身运动，不断提升广大市民身体素质。建立以居家为基础、社区为依托、机构为补充的多层次养老体系，推进医养结合，加快发展养老服务。保障妇女和未成年人合法权益，做好扶残助残工作。

大力发展教育文化事业。加快建设教育强市。积极发展学前教育，实施农村幼儿园达标工程；均衡发展义务教育，推进义务教育标准化学校建设；着力发展现代职业教育，推进职业教育三年攻坚行动计划，加快推进铁道职院、中医高专和市属三所职业院校搬迁建设；提高特殊教育普及水平，基本建成市特殊教育学校；支持发展民办教育；落实“乡村教师支持计划”。加大文化惠民力度。积极创建国家公共文化服务体系示范城市，建成神农大剧院并投入使用，启动博物馆、美术馆搬迁，不断完善公共文化服务网络。

积极创新社会治理。推进社会治理精细化、信息化、网格化，加强社会治理基础制度建设和社会信用管理，构建全民共建共享的社会治理格局。以建设“平安株洲”为目标，着力构建社会治安立体化防控体系。加快建设市监所中心。依法惩治各类违法犯罪行为，提升刑事破案率，增强群众安全感。改进信访和加强调解工作，做好社区矫正和法律援助工作，完善社会矛盾排查预警和调处化解综合机制。发挥工、青、妇等群团组织作用，落实党的民族宗教、侨务、对台政策。坚持抓安全就是抓发展的理念，完善和落实安全责任和管理制度，深入开展安全生产隐患排查治理，确保安全生产平稳可控。增强有效应对突发事件和各类事故的应急处置能力，促进社会和谐稳定。

加强国防动员和民兵预备役建设，全力支持国防和军队改革，主动帮助解决退役军人和职工的安置。进一步加强人防工作。推动军民融合发展，争创全国双拥模范城“六连冠”。

各位代表！

如期实现“三个率先”的总目标，全面完成今年各项任务，必须牢固树立以人民为中心的发展思想，自觉强化政治意识、看齐意识、带头意识，自觉践行“三严三实”，加快建设法治型、服务型、效能型和廉洁型政府，推进政府治理能力现代化。

必须依法行政。尊法学法守法用法，所有行政行为都要于法有据，把政府工作全面纳入法治轨道。用好地方立法权，发挥好立法的引领和规范作用。健全依法科学民主决策机制，严格执行《重大行政决策程序规定》。深化行政执法体制改革，严格规范公正文明执法，加快推进综合执法，全面落实行政执法责任制。自觉接受人大法律监督、工作监督和政协民主监督，认真办理人大代表建议和政协委员提案，认真听取民主党派、工商联、无党派人士和各人民团体的意见。全面推进政务公开，自觉接受群众监督，主动回应社会关切，充分体现人民意愿。

必须务实担当。经济发展进入新常态，干事创业更要有新状态。始终保持奋发有为的昂扬斗志，把推动株洲加快发展的责任扛在肩上，把群众的冷暖忧乐放在心上，主动作为，务实担当。始终保持真抓实干的工作作风，在“真”字上花力气，在“实”字上下工夫，不断完善抓落实的工作机制，把抓落实作为衡量工作成效的重要标准，营造崇尚实干的氛围，让实干在株洲蔚然成风，推动株洲各项工作干在实处、走在前列。

必须开拓创新。新常态下一些原有的行之有效的办法效力明显减弱，必须跳出思维定势，克服工作惯性，摆脱路径依赖。坚持以“五大发展”理念引领新发展，把坚持中国特色社会主义政治经济学必须把握的重大原则贯穿到实际工作中。不断创新工作方式方法，敢于向司空见惯、见怪不怪宣战。始终保持开拓创新的锐气，把创新摆在发展全局的核心位置，让创新贯穿政府的一切工作，在创新中提升能力，在创新中破解难题，在创新中培育新动力，在创新中开创新局面。

必须廉洁从政。坚持把纪律挺在前面，全面落实党风廉政建设责任制，严格执行《中国共产党廉洁自律准则》、《中国共产党纪律处分条例》。进一步完善政府投资项目、土地出让、产权交易、政府采购等制度，进一步规范公共资金、公共资源和国有资产监管，坚决打掉权力寻租空间。切实加强行政监察和审计监督，确保权力在阳光下廉洁运行。

各位代表，拼搏正当其时，圆梦适得其势。让我们在省委、省政府和市委的坚强领导下，紧紧依靠全市人民，以更加饱满的激情、更加昂扬的斗志、更加务实的作风，锐意进取、奋力拼搏，为全面实现“三个率先”总目标而努力奋斗！
